# Gestational weight gain across continents and ethnicity: systematic review and meta-analysis of maternal and infant outcomes in more than one million women

**DOI:** 10.1186/s12916-018-1128-1

**Published:** 2018-08-31

**Authors:** Rebecca F. Goldstein, Sally K. Abell, Sanjeeva Ranasinha, Marie L. Misso, Jacqueline A. Boyle, Cheryce L. Harrison, Mary Helen Black, Nan Li, Gang Hu, Francesco Corrado, Hanne Hegaard, Young Ju Kim, Margaretha Haugen, Won O. Song, Min Hyoung Kim, Annick Bogaerts, Roland Devlieger, Judith H. Chung, Helena J. Teede

**Affiliations:** 1 Monash Centre for Health Research and Implementation, Monash University, Clayton, VIC Australia; 2 Monash Diabetes and Endocrine Units, Monash Health, Locked Bag 29, Clayton Rd, Clayton, VIC 3168 Australia; 3 Kaiser Permanente, Southern California, Los Angeles, USA; 4 Tianjin Women’s and Children’s Health Center, Tianjin, China; 5 Pennington Biomedical Research Center, Baton Rouge, LA USA; 6 University Hospital, Messina, Italy; 7 Copenhagen University Hospital, Righospitalet, Copenhagen, Denmark; 8 Department of Obstetrics and Gynecology, School of Medicine, Ewha Womans University, Seoul, Republic of Korea; 9 Norwegian Institute of Public Health, Oslo, Norway; 10 Michigan State University, East Lansing, MI USA; 11 Cheil Genetal Hospital and Women’s Healthcare Centre Dankook University College of Medicine, Seoul, South Korea; 12 Department of Development and Regeneration KU Leuven, University of Leuven, Leuven, Belgium; 13 Faculty of Medicine and Health Sciences, Centre for Research and Innovation in Care (CRIC), University of Antwerp, Antwerp, Belgium; 14 Faculty of Health and Social Work, Research unit Healthy Living, UC Leuven-Limburg, Leuven, Belgium; 15 Department of Obstetrics and Gynaecology, University Hospitals KU Leuven, Leuven, Belgium; 16 Department of Obstetrics, Gynaecology and Fertility, GZA Campus Sint-Augustinus, Wilrijk, Belgium; 17 University of California, Irvine, CA USA

**Keywords:** Pregnancy, Ggestational weight gain, Maternal and infant outcomes, Obesity, Small for gestational age, Large for gestational age, Gestational diabetes, Caesarean section, Macrosomia, Preterm birth

## Abstract

**Background:**

The association between Institute of Medicine (IOM) guidelines and pregnancy outcomes across ethnicities is uncertain. We evaluated the associations of gestational weight gain (GWG) outside 2009 IOM guidelines, with maternal and infant outcomes across the USA, western Europe and east Asia, with subgroup analyses in Asia. The aim was to explore ethnic differences in maternal prepregnancy body mass index (BMI), GWG and health outcomes across these regions.

**Methods:**

Systematic review, meta-analysis and meta-regression of observational studies were used for the study. MEDLINE, MEDLINE In-Process, Embase and all Evidence-Based Medicine (EBM) Reviews were searched from 1999 to 2017. Studies were stratified by prepregnancy BMI category and total pregnancy GWG. Odds ratio (ORs) 95% confidence intervals (CI) applied recommended GWG within each BMI category as the reference. Primary outcomes were small for gestational age (SGA), preterm birth and large for gestational age (LGA). Secondary outcomes were macrosomia, caesarean section and gestational diabetes.

**Results:**

Overall, 5874 studies were identified and 23 were included (*n* = 1,309,136). Prepregnancy overweight/obesity in the USA, Europe and Asia was measured at 42%, 30% and 10% respectively, with underweight 5%, 3% and 17%. GWG below guidelines in the USA, Europe and Asia was 21%, 18% and 31%, and above was 51%, 51% and 37% respectively. Applying regional BMI categories in Asia showed GWG above guidelines (51%) was similar to that in the USA and Europe.

GWG below guidelines was associated with a higher risk of SGA (USA/Europe [OR 1.51; CI 1.39, 1.63]; Asia [1.63; 1.45, 1.82]) and preterm birth (USA/Europe [1.35; 1.17, 1.56]; Asia [1.06; 0.78, 1.44]) than GWG within guidelines. GWG above guidelines was associated with a higher risk of LGA (USA/Europe [1.93; 1.81, 2.06]; Asia [1.68; 1.51 , 1.87]), macrosomia (USA/Europe [1.87; 1.70, 2.06]; Asia [2.18; 1.91, 2.49]) and caesarean (USA/Europe [1.26; 1.21, 1.33]; Asia [1.37; 1.30, 1.45]). Risks remained elevated when regional BMI categories were applied for GWG recommendations. More women in Asia were categorised as having GWG below guidelines using World Health Organization (WHO) (60%) compared to regional BMI categories (16%), yet WHO BMI was not accompanied by increased risks of adverse outcomes.

**Conclusions:**

Women in the USA and western Europe have higher prepregnancy BMI and higher rates of GWG above guidelines than women in east Asia. However, when using regional BMI categories in east Asia, rates of GWG above guidelines are similar across the three continents. GWG outside guidelines is associated with adverse outcomes across all regions. If regional BMI categories are used in east Asia, IOM guidelines are applicable in the USA, western Europe and east Asia.

**Electronic supplementary material:**

The online version of this article (10.1186/s12916-018-1128-1) contains supplementary material, which is available to authorized users.

## Background

Gestational weight gain (GWG) is influenced by many factors including the obesogenic environment, prepregnancy body mass index (BMI), age, parity, smoking, socioeconomic status and comorbid medical conditions [1, 2]. Excess or insufficient GWG is associated with higher risks of adverse pregnancy outcomes, including preterm birth, macrosomia and caesarean delivery [3]. The US Institute of Medicine (IOM) developed GWG guidelines in 1990 and updated them in 2009 (Table 1), yet nearly three quarters of women now gain weight outside these guidelines [4, 5]. Given that lifestyle intervention improves outcomes, meeting GWG guidelines is an important target [6]. However, the IOM guidelines are based on data from primarily USA-dwelling, Caucasian and Black women, with limited ethnic diversity that may not be applicable to women from Europe and Asia. Given that Asia is the most populous continent, inhabited by 60% of the world’s population, applicability of GWG guidelines to Asian populations is an international public health priority.

**Table 1:** 2009 IOM Recommendations for gestational weight gain during pregnancy

Recommendations	Underweight	Normal weight	Overweight	Obese
Prepregnancy BMI (kg/m^2^)	< 18.5	18.5–24.9	25.0–29.9	≥30
Total weight gain range (kg)	12.5–18	11.5–16	7–11.5	5–9
Total weight gain range (lbs)	28–40	25–35	15–24	11–20

Adapted from 2009 IOM guidelines

At lower BMI, people from Asia have a greater risk for cardiovascular disease and diabetes [7, 8] than Caucasians, with a higher body fat percentage and greater central obesity [9]. During pregnancy, women from Asian countries have different risk profiles than Caucasian women. Asian-American women have a higher risk of gestational diabetes mellitus (GDM), caesarean section and low birthweight babies, and a lower risk of gestational hypertension and macrosomia compared to non-Hispanic white women [10]. Amongst Asian women, Korean and Taiwanese women have greater GWG and postpartum weight retention than women from other Asian countries [11]. In this context, GWG guidelines in Asian women may need to be considered differently; however, there is insufficient comparative research to date.

The 2009 IOM guidelines, although based on limited data, showed no ethnic differences in associations between GWG and pregnancy outcomes, whilst calling for further research [4]. Currently, there are no specific GWG guidelines for women from Asia. Most Asian studies use Caucasian-derived IOM GWG guidelines, and some use their own regional guidelines [12]. This creates heterogeneity and limits comparisons across regions, underpinning calls for new ethnic-specific regional GWG guidelines in China [13], highlighting gaps in current guidelines.

In this systematic review, meta-analysis and meta-regression, we aimed to explore ethnic differences in maternal prepregnancy BMI, GWG and health outcomes across the USA, Europe and Asia. In Asia, we also aimed to explore GWG and health outcomes using ethnic-specific regional BMI and World Health Organization (WHO) BMI categories.

## Methods

This systematic review and meta-analysis was conducted according to the Preferred Reporting Items for Systematic Reviews and Meta-Analyses (PRISMA) protocol. This protocol was registered with the PROSPERO International Prospective Register of Systematic reviews (registration number CRD42015023325). An analysis of all pooled data is published [5]. This study focused on ethnic differences in maternal BMI, GWG and maternal and neonatal outcomes.

The methods used for study eligibility, data extraction and risk of bias have been detailed previously [5] (search terms and search strategy are discussed in Additional files 1 and 2). Briefly, observational studies published in the English language between January 1999 and February 2017, with a sample size of more than 500 women were included. Studies assessing multiple pregnancies and pregnancies in women < 18 years were excluded. Inclusion required that studies present data examining the women by prepregnancy BMI category (underweight, normal weight, overweight, obese), stratified by the total pregnancy GWG (studies using weekly GWG were excluded). The odds ratio (OR) for each outcome had to be stratified by maternal BMI and GWG. Papers that mutually adjusted for BMI and GWG were excluded.

After identifying wide variations in prepregnancy BMI and GWG categories, meaningful interpretation and meta-analysis were not possible. Relevant authors were contacted to reanalyse and present data using consistent categories. Chinese and Korean studies used ethnic-specific BMI categories (China: underweight BMI < 18.5 kg/m^2^, normal weight 18.5–23.9 kg/m^2^, overweight 24–28 kg/m^2^ and obese ≥ 28 kg/m^2^; Korea: underweight BMI < 18.5 kg/m^2^, normal weight 18.5–22.9 kg/m^2^, overweight 23–25 kg/m^2^ and obese ≥ 25 kg/m^2^) whilst Japanese and Taiwanese studies used WHO BMI categories (underweight < 18.5 kg/m^2^, normal weight 18.5–24.9 kg/m^2^, overweight 25–29.9 kg/m^2^ and obese ≥30 kg/m^2^).

Primary outcomes were (1) small for gestational age (SGA): < 10th percentile of birthweight for sex and gestational age, (2) pre-term birth: spontaneous birth < 37 weeks gestation, (3) large for gestational age (LGA): > 90th percentile of birthweight for sex and gestational age. Secondary outcomes were (1) macrosomia: birthweight > 4000 g, (2) caesarean section and (3) GDM.

### Strategy for data synthesis

Study findings were synthesised based on target population characteristics, type of study and outcome. Proportions were calculated using the pooled number in a group divided by the total number (%). The chi-squared test was used to assess difference in proportion of women within BMI categories and GWG categories between regions. The two-sample test of proportions was used to assess differences between two particular regions.

Summaries of outcomes associated with GWG were produced for each study by calculating the ORs and 95% confidence intervals (CIs), using the recommended GWG within each BMI category as the reference. Where two or more studies assessed the same outcome, the results were pooled using random-effects meta-analysis, calculating the OR and 95% CI for each outcome. Extracted pooled ORs for each outcome were combined to construct a summary pooled OR for all outcomes. Crude data was used where possible given the variation in control for confounding factors. However, some papers presented adjusted ORs only [14–21]. US and European studies were combined as one group in the meta-analysis of pregnancy outcomes (to allow for two or more studies to assess each outcome) and compared to Asian studies. We were unable to demonstrate statistical significance for comparison of ORs for SGA, preterm birth, LGA, macrosomia and caesarean section between the US/Europe and Asian studies due to similar ORs and overlap in CIs.

Heterogeneity was assessed using the *I*^2^ statistic. An *I*^2^ value greater than 50% was indicative of substantial heterogeneity [22] . Where there was sufficient data available, a meta-regression analysis was performed to investigate sources of heterogeneity, including percentage of smokers in pregnancy, mean age and percentage nulliparity. Sufficient data on race/ethnicity was not available for inclusion in the meta-regression. Studies from Europe and Asia did not provide information regarding race or ethnicity. Studies from the USA provided race/ethnicity data; however, this varied with reporting methods (some report percentage of total population, others report percentage stratified by GWG).

A further analysis of women living in Asian countries was performed comparing studies using regional BMI categories (Chinese and Korean studies) and WHO BMI categories (Japanese and Taiwanese) assessing alignment with 2009 IOM GWG guidelines and maternal and infant adverse outcomes. Statistical analysis used Stata software v.14 and was supported by a biostatistician (SR).

## Results

**Fig. 1:**
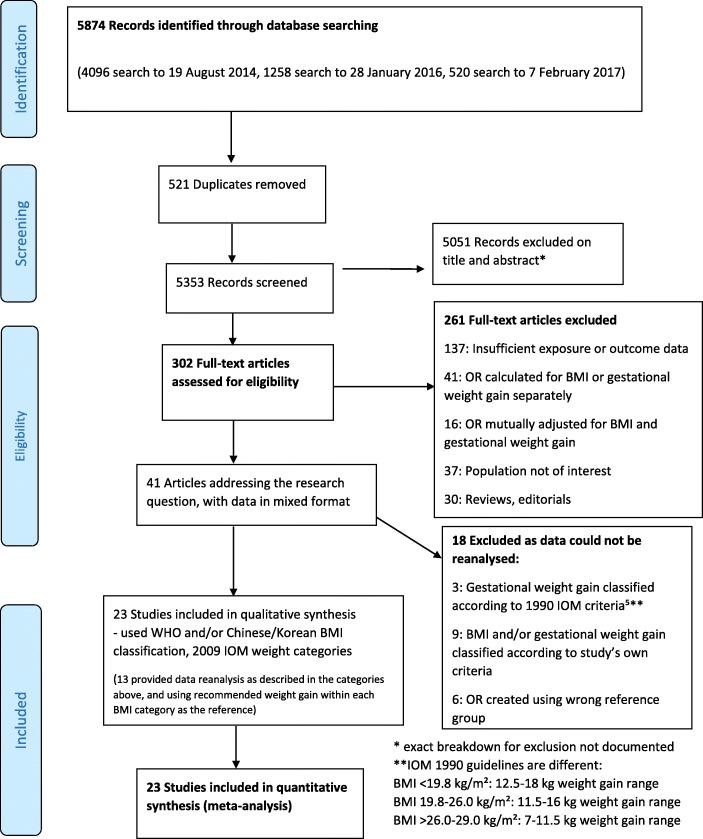
Flow Diagram of study selection process

From 5874 studies identified by the initial search, 302 studies were selected for full text review (Fig. 1) and 261 studies were excluded, using a priori selection criteria. Forty papers grouped women by prepregnancy BMI category (underweight, normal weight, overweight, obese), stratified by the total GWG for the pregnancy. One study [23] did not initially meet inclusion criteria because ORs were not stratified by both BMI and GWG. However, through collaborations, this data was available in the required format. Where required, authors were contacted for data reanalysis, and 13 collaborated (Additional file 2).

In total, 23 cohort studies [12, 14–21, 23–36] were included in this systematic review and meta-analysis, reporting data on more than 1 million women (*n* = 1,309,136).

### Study characteristics

Table 2 describes the study design and size, eligibility criteria and outcomes (descriptive characteristics are shown in Additional file 3: Table S1). Eighteen studies were retrospective, five were prospective [14, 25, 28, 31, 36]. Ten studies were from the USA [14, 17, 18, 20, 23, 27, 29, 30, 32, 33], five from western Europe (one each from Norway [25], Belgium [35], Italy [24], Denmark [28] and Sweden [15]) and eight from east Asia (four from China [16, 26, 31, 36], two from Korea [12, 34], one each from Taiwan [21] and Japan [19]). The sample size ranged from 1034 to 570,672.

**Table 2:** Characteristics of 23 included studies

Study	Country	Study period	Study design	Sample size	Setting	Outcomes
Durst, 2016 [20]	USA	2000–2014	Retrospective	5651	University of Alabama, Birmingham	SGA, LGA, macrosomia, caesarean section
Enomoto, 2016 [19]	Japan	2013	Retrospective	97,157	Japan Society of Obstetrics and Gynaecology Registry system with 280 participating hospitals	SGA, preterm birth, LGA, macrosomia, caesarean
Hung, 2016 [21]	Taiwan	2009–2015	Retrospective	10,973	Taipei Chang Gung Memorial Hospital	SGA, LGA, macrosomia, caesarean section
Xiong^e^, 2016 [36]	China	2012–2013	Prospective	57,891	Hospitals and community centres	Caesarean section
Bogaerts, 2015 [35]	Belgium	2009–2011	Retrospective	18,053	Flemish study center for perinatal	Caesarean section, macrosomia, LGA
Shin, 2015 [33]	USA	2004–2011	Retrospective	219,868	Pregnancy risk assessment monitoring system (PRAMS)	Preterm birth, SGA, LGA
Wen^e^, 2015 [16]	China	2009–2013	Retrospective	13,776	Jishuitan Hospital	Preterm birth
Yang^e^, 2015 [31]	China	2011–2013	Prospective	85,765	Wuhan Women and Children Health Care Center	Macrosomia
Badon, 2014 [14]	USA	2000–2006	Prospective	5297	North American Field Centers, HAPO	LGA
Chihara, 2014 [17]	USA	2003–2005	Retrospective	19,130	Hawaii’s special supplemental program for women, infants and children (WIC)	Macrosomia
Haugen^c^, 2014 [25]	Norway	1999–2008	Prospective	56,082	Norwegian Mother and Child cohort study	Macrosomia, caesarean section
Lee^d^, 2014 [34]	Korea	2010–2012	Retrospective	16,297	Single medical centre	LGA
Swank, 2014 [32]	USA	2007	Retrospective	1034	Californian birth certificate data	Caesarean, macrosomia
Black, 2013 [23]	USA	2005–2010	Retrospective	9835	Kaiser Permanente Southern California	LGA (provided additional outcomes in reanalysis incl. SGA, preterm, macrosomia and caesarean section
Kominiarek^b^, 2013 [18]	USA	2002–2008	Retrospective	21,020	12 institutions (19 hospitals)	Caesarean section, SGA, LGA, macrosomia
Li^a^, 2013 [26]	China	2009–2011	Retrospective	33,973	Tianjin Women’s and Children’s Health Centre	Caesarean, preterm delivery, LGA, SGA macrosomia
Di Benedetto, 2012 [24]	Italy	2004–2009	Retrospective	2225	University Hospital	Macrosomia, caesarean section
Moore Simas, 2012 [29]	USA	2006–2010	Retrospective	11,203	University Hospital	SGA, LGA
Blomberg, 2011 [15]	Sweden	1993–2008	Retrospective	46,595	Swedish Medical birth registry	Caesarean, LGA, SGA
J Park^d^, 2011 [12]	Korea	2005–2007	Retrospective	2311	University Hospital	SGA, LGA, macrosomia, caesarean section, preterm birth
S Park, 2011 [27]	USA	2004–2007	Retrospective	570,672	Florida birth certificate data	SGA, LGA
Vesco, 2011 [30]	USA	2000–2005	Retrospective	2080	Kaiser Permanente group practice	Macrosomia, LGA, SGA
Rode, 2007 [28]	Denmark	1996–1998	Prospective	2248	University Hospital	Macrosomia

^a^Data according to both Chinese and WHO BMI categories (Chinese used here)

^b^Sample size changed when provided additional data, OR not recalculated

^c^Sample size changed when provided additional data

^d^Data according to both Korean and WHO BMI categories (Korean used here)

^e^Data according to Chinese BMI categories

*HAPO* Hyperglycaemia and Adverse Pregnancy Outcomes, *NR* not reported

Overall, 66% (*n* = 865,790) of women were from the USA, 10% (*n* = 125,203) from Western Europe and 24% (*n* = 318,143) from east Asia.

### Analysis by region: USA, Europe and Asia

In the descriptive analysis of maternal BMI only, it was required to exclude two European studies [15, 35] (52% of European women) and four US studies [18, 20, 30, 32] (3% of US women) which studied obese women only, and one Asian study [16] (4% of Asian women) which studied normal weight women only. In the remaining studies, overweight and obesity were present in 43% of women in the USA, 31% in Europe and 10% in Asia (Table 3). Underweight BMI was present in 5% in the USA, 3% in Europe and 17% in Asia. The proportion of women within each BMI category was different between the regions (*p* < 0.0001) (using the chi-squared test).

**Table 3:** Body mass index prepregnancy by regions (%)

Region	Underweight	Normal weight	Overweight	Obese
Including all studies				
USA	5	51	23	21
Europe	1	33	10	6
Asia	16	74	8	2
Excluding studies that assessed obese women [15, 18, 20, 30, 32, 35] or normal weight women only [16]				
USA	5	53	24	18
Europe	3	67	21	9
Asia (overall)	17	73	8	2
Asia (regional BMI)	16	73	9	2
Asia (WHO BMI)	18	71	8	3

Overall, underweight women had the greatest prevalence of GWG below guidelines (43%), whereas overweight women, followed by obese women, had the greatest prevalence of GWG above guidelines (64% and 60% respectively) (Table 4).

**Table 4:** Proportions of women gaining below, within and above guidelines, stratified by prepregnancy BMI (%)

BMI group	Below IOM	Within IOM	Above IOM
Underweight	43	36	21
Normal weight	28	36	36
Overweight	13	23	64
Obese	19	21	60

Data from 20/23 studies: *n* = 1,146,350 (88% of total population). Excluding studies that did not stratify GWG by BMI category [17, 31, 36]

For GWG below guidelines, prevalence was 21%, 18% and 31% in the USA, Europe and Asia respectively, including all Asian data (Table 5). The proportion of women gaining below guidelines was different between the three regions (*p* < 0.0001) (using the chi-squared test).

**Table 5:** Gestational weight gain during pregnancy by regions (%)

Region	Below guidelines	Within guidelines	Above guidelines
Including all studies			
USA	21	28	51
Europe	18	31	51
Asia (overall)	31	32	37
Asia (regional BMI)	16	33	51
Asia (WHO BMI)	60	31	9

Including all studies

For GWG above guidelines, prevalence was 51%, 51% and 37% in the USA, Europe and Asia respectively, including all Asian data. The proportion of women above guidelines was different between the three regions (*p* < 0·0001) (using the chi-squared test). GWG above guidelines was higher in the USA than Asia (*p* < 0·0001) and higher in Europe than Asia (*p* < 0·0001), but this was not true between the USA and Europe (*p* = 1·0) (using the two-sample test of proportions).

However, when Asian studies applying regional BMI categories only were analysed, GWG above guidelines (51%) was no longer significantly different from GWG above guidelines in the USA and Europe (*p* = 0.28). There was a substantial difference between GWG below guidelines in Asia, using regional BMI (16%), compared to WHO BMI categories when applying IOM guidelines (60%).

**Fig. 2:**
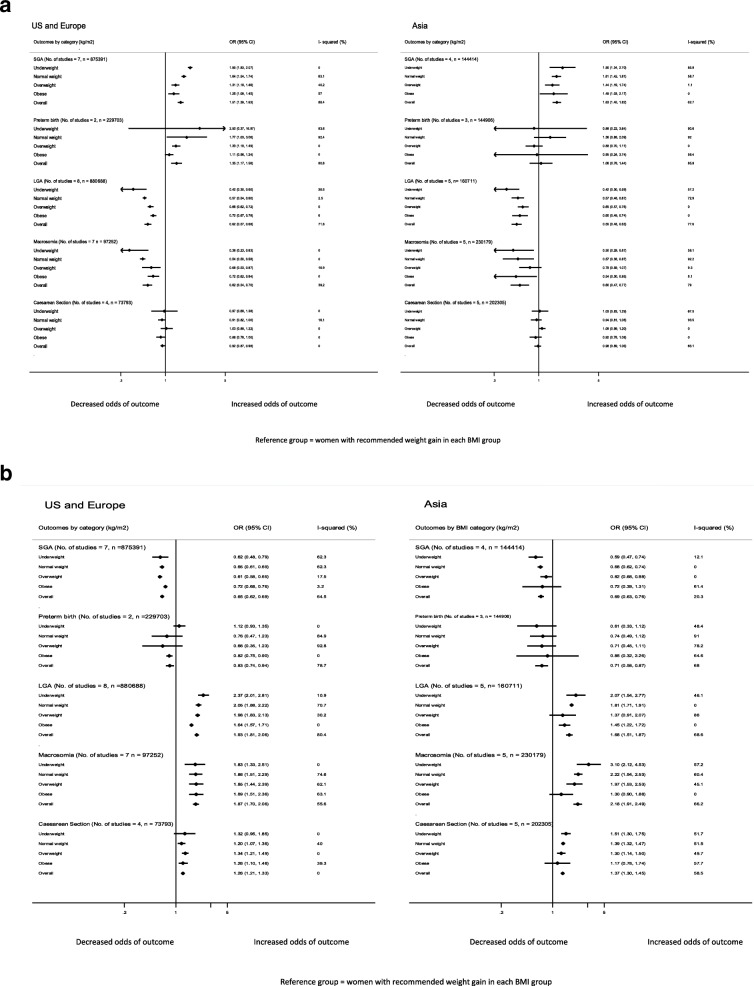
**a** Pooled odds ratio for individual outcomes for USA and Europe combined vs Asia, for the association between GWG below guidelines with adverse outcomes. **b** Pooled odds ratio for individual outcomes for USA and Europe combined vs Asia, for the association between GWG above guidelines with adverse outcomes

A summary of pooled ORs for primary and secondary outcomes is given in Fig. 2a and b and Table 6. Pooled ORs for individual analyses for outcomes are presented in Additional file 4.

**Table 6:** Odds ratios for pregnancy outcomes by regions

Outcome	USA and Europe		Asia overall		Regional BMI studies (Chinese and Korean)		WHO BMI studies (Japanese and Taiwanese)	
	GWG < guidelines	GWG > guidelines	GWG < guidelines	GWG > guidelines	GWG < guidelines	GWG > guidelines	GWG < guidelines	GWG > guidelines
SGA	1.51 (1.39, 1.63)	0.65 (0.62, 0.69)	1.63 (1.45, 1.82)	0.69 (0.63, 0.76)	1.43 (1.2, 1.7)	0.65 (0.57, 0.75)	1.77 (1.56, 2.01)	0.70 (0.63, 0.79)
Preterm birth	1.35 (1.17, 1.56)	0.83 (0.74, 0.94)	1.06 (0.78, 1.44)	0.71 (0.58, 0.87)	N/A	N/A	N/A	N/A
LGA	0.62 (0.57, 0.68)	1.93 (1.81, 2.06)	0.55 (0.48, 0.63)	1.68 (1.51, 1.87)	0.61 (0.48, 0.76)	1.86 (1.66, 2.09)	0.49 (0.39, 0.62)	1.49 (1.18, 1.87)
Macrosomia	0.62 (0.54, 0.70)	1.87 (1.70, 2.06)	0.60 (0.47, 0.77)	2.18 (1.91, 2.49)	0.75 (0.68, 0.83)	2.0 (1.71, 2.34)	0.52 (0.31, 0.88)	2.76 (2.25, 3.38)
Caesarean section	0.92 (0.87, 0.98)	1.26 (1.21, 1.33)	0.98 (0.89, 1.06)	1.37 (1.30, 1.45)	0.94 (0.85, 1.04)	1.43 (1.34, 1.52)	1.02 (0.92, 1.14)	1.32 (1.19, 1.46)

*N/A* Unable to perform meta-analysis for preterm birth because less than 2 studies within each region

#### Primary outcomes

##### SGA: eleven studies (seven USA/Europe; four Asia)

Eleven studies assessed SGA. This was defined as birthweight < 10th percentile for gestational age in five studies [12, 19, 26, 27, 33]; four additionally accounted for sex [21, 23, 25, 29], one for sex and race/ethnicity [30] and another for sex, race and parity [20].

GWG below guidelines was associated with a higher risk for SGA than GWG within guidelines; for USA/Europe OR 1.51 (1.39–1.63), *I*^2^ = 88% and for Asia OR 1.63 (1.45–1.82), *I*^2^ = 63. The association of SGA risk was highest with underweight women for both USA/Europe (1.95; 1.83–2.07) and Asia (1.90; 1.34–2.70).

GWG above guidelines was associated with lower risk for SGA than GWG within guidelines: USA/Europe (OR 0.65; 0.62–0.69) *I*^2^ = 65% and Asia (OR 0.69; 0.63–0.76) *I*^2^ = 20%.

##### Preterm birth: five studies (two USA/Europe; three Asia)

Five studies assessed preterm birth (< 37 weeks gestation); four did not specify whether this was spontaneous or induced [16, 23, 26, 33] and one specified spontaneous and induced combined [19].

GWG below guidelines was associated with a higher risk for preterm birth than GWG within guidelines: USA/Europe (OR 1.35; 1.17–1.56) *I*^2^ = 81% and Asia (OR 1.06; 0.78–1.44) *I*^2^ = 86%.

GWG above guidelines was associated with a lower risk for preterm birth than GWG within guidelines: USA/Europe (0.83; 0.74–0.94) *I*^2^ = 79% and Asia (OR 0.71; 0.58–0.87) *I*^2^ = 68%.

##### LGA: thirteen studies (eight USA/Europe; five Asia)

Thirteen studies assessed LGA. This was defined as birthweight > 90th percentile for gestational age in six studies [12, 19, 26, 27, 33, 34]. Four defined LGA by additionally accounting for infant sex [21, 23, 25, 29], one for sex and race/ethnicity [30], one for sex, race and parity [20] and one for sex, parity and study centre [14].

GWG below guidelines was associated with a lower risk for LGA than GWG within guidelines: USA/Europe (OR 0.62; 0.57–0.68) *I*^2^ = 72% and Asia (OR 0.55; 0.48–0.63) *I*^2^ = 78%. The risk was lowest in the underweight women: (USA/Europe [OR 0.42; 0.30–0.60] and Asia [OR 0.42; 0.30–0.59]).

GWG above guidelines was associated with a higher risk for LGA: USA/Europe (OR 1.93; 1.81–2.06) *I*^2^ = 80% and Asia (OR 1.68; 1.51–1.87) *I*^2^ = 69%. For both groups, the risk was greatest in underweight women, with risk decreasing as BMI increased.

#### Secondary outcomes

##### Macrosomia: twelve studies (seven USA/Europe; five Asia)

Macrosomia was defined as birthweight > 4000 g in the majority [12, 17, 19–21, 23–26, 28, 31]; one study used birthweight > 4500 g [30].

GWG below guidelines was associated with a lower risk for macrosomia than GWG within guidelines: USA/Europe (OR 0.62; 0.54–0.70) *I*^2^ = 39% and Asia (OR 0.60; 0.47–0.77) *I*^2^ = 79%.

GWG above guidelines was associated with a higher risk for macrosomia: USA/Europe (OR 1.87; 1.70–2.06) *I*^2^ = 56% and Asia (OR 2.18; 1.91–2.49) *I*^2^ = 66%. In Asia, the risk decreased as the BMI increased.

##### Caesarean section: nine studies (four USA/Europe; five Asia)

Nine studies assessed caesarean section. Seven included emergency and elective deliveries [12, 19, 23–26, 36] and two did not specify [20, 21]. Two [20, 23] included repeat caesarean (total caesarean section), one primary caesarean only [21] and six did not distinguish these.

GWG below guidelines was associated with a lower risk for caesarean: USA/Europe (OR 0.92; 0.87–0.98) *I*^2^ = 0%, with no statistically significant result for Asia (OR 0.98; 0.89–1.06) *I*^2^ = 83%.

GWG above guidelines was associated with a higher risk for caesarean: USA/Europe (OR 1.26; 1.21–1.33) *I*^2^ = 0% and Asia (OR 1.37; 1.30–1.45) *I*^2^ = 59%. In Asia, the risk was greatest in underweight women (OR 1.51; 1.30–1.45).

##### Gestational diabetes: Six studies

Six studies assessed GDM, but did not use consistent definitions, and had different findings for GWG above guidelines and GDM risk, preventing the intended meta-analysis of GDM and its relationship to GWG.

We were unable to demonstrate statistical significance for comparison of ORs for SGA, preterm birth, LGA, macrosomia and caesarean section between the USA/Europe and Asian studies due to similar ORs and overlap in CIs.

#### Subgroup analysis: Asian studies

Of the eight studies from Asia, four were from China [16, 26, 31, 36], two from Korea [12, 34], with one each from Japan [19] and Taiwan [21].

Results are stratified by country in Additional files 5 and 6 (Table S2: BMI at onset of pregnancy and Table S3: GWG during pregnancy).

#### Comparison between studies using ethnic-specific regional BMI categories and WHO BMI categories

A further analysis comparing studies using regional BMI categories (Chinese and Korean studies) and WHO BMI categories (Japanese and Taiwanese studies) was performed to assess for differences in adherence to 2009 IOM GWG guidelines and differences in maternal and infant adverse outcomes.

Asian studies using ethnic-specific regional BMI categories showed 16% of women with GWG below guidelines, 33% within and 51% above, whereas studies using WHO BMI categories had 60% with GWG below, 31% within and 9% above (Table 5).

An additional meta-analysis was performed in Asian studies, where studies using regional BMI categories (Chinese and Korean studies) were compared to those studies using WHO BMI categories (Japanese and Taiwanese studies) (Table 6). Pooled ORs for individual analyses for outcomes are presented in Additional file 7.

SGA, LGA, macrosomia and caesarean section could be examined in a meta-analysis (Table 6).

Wen et al. only included normal weight women, and Yang et al. had women in all weight categories except obese. Yang defined underweight as < 18 kg/m^2^.

For OR calculation, Hung, Xiong and Yang combined overweight and obese into one group. The OR was used for the overweight group here. Although Enomoto created separate ORs for overweight and obese, only overweight was used in the meta-analysis as there were no comparison groups for obese.

### Meta-regression

Substantial heterogeneity (*I*^2^ > 50%) was present for GWG below guidelines for SGA (USA/Europe and Asia), preterm birth (USA/Europe and Asia), LGA (USA/Europe and Asia), macrosomia (Asia) and caesarean section (Asia), and for GWG above guidelines for SGA (USA/Europe), preterm birth (USA/Europe and Asia), LGA (USA/Europe and Asia), macrosomia (USA/Europe and Asia) and caesarean section (Asia).

Where there was sufficient data available, we performed a meta-regression analysis to investigate possible sources of heterogeneity, including percentage of smokers in pregnancy, mean age and percentage nulliparity (Additional file 8) in studies from the USA/Europe and Asia.

The effect of GWG below guidelines on SGA (*p* < 0.0001) for USA/Europe was associated with mean maternal age (*p* < 0.0005) and nulliparity (*p* < 0.0005) and marginally associated with smoking (*p* = 0.056). The GWG below guidelines effect on LGA (*p* = 0.002) for USA/Europe was associated with mean maternal age (*p* = 0.021) and nulliparity (*p* < 0.005). The effect of GWG above guidelines on LGA was significantly associated with nulliparity (*p* = 0.025) and marginally associated with mean age (*p* = 0.084) for the USA/Europe. Heterogeneity was unexplained for the remaining outcomes.

### Publication bias

There was no evidence of publication bias for SGA, LGA, macrosomia or caesarean section (Additional file 9). Assessment for publication bias was not assessed for preterm birth (less than five studies).

### Risk of bias

Participants were selected from maternity clinics or from large datasets (Additional file 10). Apart from two studies [17, 31], there was adequate description of inclusion and exclusion criteria. Studies were mostly retrospective, with three prospective studies [14, 25, 28] and one unspecified [31]. Given the nature of observational studies, attrition bias was not considered relevant. Performance bias was difficult to assess. Very few studies provided information regarding diet/exercise advice given and whether this differed between groups. The overweight and obese women may have been treated more intensively, and this could be a source of bias. However, we postulate this difference would be similar across studies and therefore propose that studies carry a low risk of performance bias overall.

There were three studies with moderate risk of bias and 16 studies with low risk of bias. Main reasons for moderate risk of bias included self-reported final weight (detection bias), self-reported outcome measures (detection bias), failure to report all outcomes (report bias) and insufficient adjustment for confounding variables (confounding bias). Authors on 15 studies reported no conflict of interest.

## Discussion

In this study of 1,309,136 pregnancies, we present a systematic review, meta-analysis and meta-regression incorporating women from diverse ethnicities across three continents, contemporary cohorts and from across the BMI range. We explore ethnic differences in prepregnancy BMI, prevalence of GWG outside IOM guidelines and maternal and neonatal health outcomes between women living in the USA, western Europe and east Asia. Within Asia, we compare studies applying regional and WHO BMI categories. Women in the USA and Europe have higher prepregnancy BMI, higher prevalence of GWG above guidelines and lower rates of GWG below guidelines than women in Asia. However, when applying regional BMI categories, women in Asia have similar GWG above guidelines to the other continents, but retain lower prevalence of GWG below guidelines. GWG outside guidelines is associated with adverse health outcomes across all regions. A greater percentage of women in Asia had GWG below guidelines, using WHO BMI (60%) compared to regional BMI categories (16%), yet WHO BMI was not accompanied by increased risks of adverse outcomes.

Given that Asian women have greater risks of health complications at a lower BMI, Asian countries often use lower BMI cut-offs for overweight and obese categories. However in 2004, a WHO review of relevant evidence concluded there was no clear cut-off for overweight and obesity for those of Asian ethnicity, and thus WHO did not change their current BMI guidelines [37]. They did, however, identify trigger points of > 23 kg/m^2^ and > 27.5 kg/m^2^, representing increased and high risks respectively for public health action. In practice, BMI categories commonly used in China [16, 26, 31] are underweight BMI < 18.5 kg/m^2^, normal weight 18.5–23.9 kg/m^2^, overweight 24–28 kg/m^2^ and obese ≥28 kg/m^2^. In Korea, the classifications are underweight BMI < 18.5 kg/m^2^, normal weight 18.5–22.9 kg/m^2^, overweight 23–25 kg/m^2^ and obese ≥25 kg/m^2^ [12, 34]. Studies from Taiwan [38, 39] and Japan used WHO BMI categories [40] despite Japanese Society of Obesity guidelines that define obesity at a BMI ≥ 25 kg/m^2^ [41]. The European Board and College of Obstetrics and Gynaecology (EBCOG) [42] notes difficulties in accurately comparing prevalence of prepregnancy BMI groups internationally with heterogeneity of data sets. However, comparison is important across regions to inform our understanding of relationships between GWG and pregnancy outcomes. To the best of our knowledge, this is the only systematic review comparing prepregnancy BMI and exploring relationships to GWG and health outcomes across international settings. We have compared Asian studies using regional and WHO BMI categories in assessment of prepregnancy BMI, GWG and pregnancy health outcomes to explore applicability of regional and WHO BMI categories in applying IOM GWG guidelines.

Applying WHO prepregnancy BMI categories, the USA had the greatest prevalence of overweight and obesity at 43%, consistent with trends from the 2013–2014 National Health and Nutrition Examination Survey (NHANES), with 37% of reproductive-aged women obese [43]. This is significant as, preconception, a higher BMI independently increases pregnancy complications including GDM, preeclampsia, caesarean section and LGA [44, 45]. In contrast, Asia had the greatest prevalence of women in normal weight and underweight categories. A lower BMI preconception is associated with increased risks including SGA [46]. The high prevalence of prepregnancy BMI outside of the healthful range shown here highlights the critical need to focus on achieving healthy preconception weight, especially in the USA, but also across Europe and Asia.

Women in the USA and Europe had higher GWG above guidelines than women in Asia. However, in studies applying ethnic-specific regional BMI categories, women in Asia had similar rates of GWG above guidelines. The prevalence of GWG above guidelines is consistent with observational studies [47–50]. Excess GWG increases adverse pregnancy outcomes, independent of BMI, as demonstrated here, and also increases postpartum weight retention and obesity [45, 51]. A systematic review of postpartum weight retention in Asian women found that whilst prepregnancy BMI had an impact, GWG was the most important predictor [11], supporting the clinical relevance of our findings on long-term contribution to obesity. Here we have advanced the literature to highlight the high prevalence of GWG above guidelines across the USA, Europe and Asia and show the impact of using regional BMI categories on the application of IOM guidelines.

Exploring health outcomes by GWG, we combined USA and Europe to ensure adequate numbers for meta-analysis and compared USA/Europe to Asia. Across regions, GWG below guidelines was associated with a higher risk of SGA and preterm birth, compared to GWG within guidelines. Likewise across regions, GWG above guidelines was associated with a greater risk for LGA, macrosomia and caesarean section. For women in Asia, adverse outcomes were noted applying both regional and WHO BMI categories. We were generally unable to compare differences in adverse health outcomes because ORs between regions were similar with overlapping CIs. Further research using both regional and WHO BMI categories in all studies of GWG and health outcomes may be useful. We also support the recommendations for standardisation of GWG categories and core outcome parameters to enable more accurate comparisons for future studies [42, 52].

With high prepregnancy BMI, high rates of GWG above guidelines and clear adverse health outcomes shown here across the USA, Europe and Asia, and in our pooled data analysis [5], intervention is clearly vital. The Journal of the American Medical Association editorial accompanying our recent data analysis on GWG discussed barriers to healthful lifestyle intervention during pregnancy in addressing GWG and improving health outcomes [53]. Barriers included inadequate evidence of improvement of adverse pregnancy outcomes and modest changes in GWG. Yet, the largest individual patient data (IPD) analysis of 36 randomised controlled trials in pregnancy (~ 12,000 women) [6], recently published in *The BMJ*, demonstrates that even modest reduction in excessive GWG improves outcomes, reducing caesarean section, preterm birth and GDM, the latter being particularly modifiable with physical activity intervention. Reported results were independent of maternal characteristics including age, BMI, parity and ethnicity, enhancing generalisability of the findings. It appears that even modest changes to lifestyle and GWG effectively reduce adverse health outcomes, affirming the need for implementation of healthful lifestyle in routine antenatal care for public health impact [54].

There may also be differences to consider within Asia. Comparing Asian studies, prepregnancy BMI was similar. Overall, 16% of Chinese women were underweight, 74% normal weight and 9% overweight and obese. These values are lower than those of recent cohort studies, where 15–28% of reproductive-aged women in China are above healthy weight [13, 47]. This contrasted with Japan, with 18%, 71% and 11% respectively. In China 53% of women gained above GWG guidelines consistent with the USA and Europe. In Japan GWG below guidelines was 64%, with only 7% above. These differences arguably occur because WHO BMI categories were applied in Japan. Differences may also relate to ethnic variation. In Singapore, difference in GWG between ethnicities was postulated to be due to difference in diet quality and psychosocial factors [55]. However we postulate that the degree of observed difference primarily related to application of BMI categories. Asian studies have already suggested the need for specific guidelines [56]. In 2000, Chinese-specific guidelines for GWG [57] were developed, but have not been commonly adopted, with most Asian studies using mainly Caucasian-derived IOM GWG guidelines [55]. A call has been made for multi-centre collaboration to create optimal GWG guidelines for Asian women using modified BMI categories [58]. Here however, we demonstrate that applying regional BMI categories generated GWG patterns and health outcomes similar to those in the USA/Europe. With regional BMI categories, apparent higher risks of macrosomia and caesarean section were demonstrated. Overall our data are reassuring for clinicians and policy makers that IOM GWG guidelines are applicable in women of Asian background, provided regional BMI categories are used, to avoid overestimation of GWG below recommendations that are not accompanied by increased risks of adverse outcomes.

Limitations of our study include the lack of cohorts from developing countries and the exclusion of non-English language articles. It did not include studies from eastern Europe and south Asian countries, which have historical and ethnic differences from adjacent western European and east Asian countries respectively, yet this is the broadest systematic review and meta-analysis performed to date. For the meta-analysis, we combined the USA and Europe into one group, due to inadequate reported outcomes. Within each study there may be heterogeneity regarding race/ethnicity, and results should not be interpreted that the sample represented the country of origin. The European and Asian studies did not provide demographic data, and we have assumed the populations in these studies to be largely homogeneous. Studies from the USA do include some women from Asia, and where reported proportions are small, reporting is inconsistent, limiting capacity to interpret the overall prevalence of Asian women in US and European studies. Preterm birth was not adjusted for gestational age, potentially resulting in less total gestational weight gain than would have been otherwise attained. Meta-analyses for GDM could not be performed due to deficiencies in the primary data sets. Heterogeneity among studies may affect the reliability of the results, although this was only relevant for the effect of GWG below guidelines in SGA and LGA in USA/Europe. Lastly, we included studies published before 2009 IOM guidelines, so treating physicians and midwives may have had different GWG targets and guidelines compared to studies from after that time.

Strengths are the inclusion of common maternal and infant risks associated with GWG below and above the IOM 2009 guidelines across the entire prepregnancy BMI spectrum, with an analysis across three continents. Notably, a quarter of the women in these studies were from Asia. This is the only systematic review that has compared Asian studies applying regional compared to WHO BMI categories. We searched four databases, performed a thorough risk of bias appraisal and sought international collaboration to facilitate reanalysis, enabling broad inclusion of data in excess of 1.3 million pregnant women. The collaboration with authors has enabled data in a more homogeneous format for meta-analysis, with unprecedented data integration and meta-analysis.

## Conclusions

In this study of 1,309,136 pregnancies, incorporating women from diverse ethnicities, contemporary cohorts and from across the BMI range, we show that women from the USA and Europe have higher prepregnancy BMI than those from Asia (even when applying regional BMI categories). In the USA and Europe, GWG above guidelines appeared higher than in Asia and GWG below guidelines was highest in Asia. However in Asian studies applying regional BMI categories, GWG above guidelines was similar across the USA, Europe and Asia. In Asia, regional BMI categories may be more applicable than WHO BMI categories when applying IOM GWG guidelines. Across all prepregnancy BMI categories and in different ethnicities, insufficient GWG is associated with increased risk of SGA and preterm birth and excess GWG with increased risk of LGA, macrosomia and caesarean section. Risks associated with excess GWG may be higher in women from Asia. These findings have practice and policy implications. This work attests to the broad applicability of the 2009 IOM guidelines, when Asian regional BMI categories are applied. As lifestyle interventions in pregnancy increase attainment of recommended GWG and show health benefits, IOM implementation of GWG guidelines and pregnancy lifestyle interventions should be considered broadly across maternity care [59, 60].

## Additional files

Additional file 1: Search terms. (DOCX 17 kb)

Additional file 2: Additional methods. (DOCX 14 kb)

Additional file 3: **Table S1.** Descriptive characteristics of 23 included studies. (DOCX 27 kb)

Additional file 4: **Figure S1.** Summary of pooled OR for the association between gestational weight gain below and above guidelines for adverse outcomes. (DOCX 220 kb)

Additional file 5: **Table S2.** Body mass index at onset of pregnancy for Asian studies. (DOCX 17 kb)

Additional file 6: **Table S3.** Gestational weight gain during pregnancy for Asian studies. (DOCX 14 kb)

Additional file 7: **Figure S2.** Asian subgroup analysis: studies using local BMI categories (China, Korea) vs WHO BMI categories (Japan, Taiwan). Summary of pooled OR for the association between gestational weight gain below and above guidelines for adverse outcomes. (DOCX 106 kb)

Additional file 8: **Table S4.** Meta-regression. (DOCX 21 kb)

Additional file 9: **Figure S3.** Publication bias. (DOCX 53 kb)

Additional file 10: **Table S5.** Summary of risk of bias assessment. (DOCX 20 kb)

## Abbreviations

BMI: Body mass index
GDM: Gestational diabetes mellitus
GWG: Gestational weight gain
IOM: Institute of Medicine
LGA: Large for gestational age
SGA: Small for gestational age
WHO: World Health Organization
