# Supplementary material for: Gestational weight gain across continents and ethnicity: systematic review and meta-analysis of maternal and infant outcomes in more than one million women
Source: BMC Med. 2018 Aug 31;16:153. doi: 10.1186/s12916-018-1128-1 (PMC6117916; doi:10.1186/s12916-018-1128-1)
Supplement: Supplementary file 1 — Search terms. (DOCX 17 kb) [file 12916_2018_1128_MOESM1_ESM.docx]

| **Additional file 1: Search terms**  **Searches** | **Results** |
| --- | --- |
| Weight Gain/ | 23347 |
| Pregnancy/ | 702831 |
| 1 and 2 | 3146 |
| (weight and gain).mp. [mp=title, abstract, original title, name of substance word, subject heading word, keyword heading word, protocol supplementary concept word, rare disease supplementary concept word, unique identifier] | 57991 |
| (weight and change).mp. [mp=title, abstract, original title, name of substance word, subject heading word, keyword heading word, protocol supplementary concept word, rare disease supplementary concept word, unique identifier] | 48999 |
| 4 or 5 | 100281 |
| pregnan*.mp. [mp=title, abstract, original title, name of substance word, subject heading word, keyword heading word, protocol supplementary concept word, rare disease supplementary concept word, unique identifier] | 775673 |
| gestation*.mp. [mp=title, abstract, original title, name of substance word, subject heading word, keyword heading word, protocol supplementary concept word, rare disease supplementary concept word, unique identifier] | 176589 |
| 7 or 8 | 822868 |
| 6 and 9 | 12406 |
| 3 or 10 | 12406 |
| diabetes, gestational/ or fetal macrosomia/ | 7179 |
| (gestational and diab*).mp. [mp=title, abstract, original title, name of substance word, subject heading word, keyword heading word, protocol supplementary concept word, rare disease supplementary concept word, unique identifier] | 11182 |
| gdm.mp. [mp=title, abstract, original title, name of substance word, subject heading word, keyword heading word, protocol supplementary concept word, rare disease supplementary concept word, unique identifier] | 2715 |
| Pre-Eclampsia/ | 24030 |
| pre-eclamp*.mp. [mp=title, abstract, original title, name of substance word, subject heading word, keyword heading word, protocol supplementary concept word, rare disease supplementary concept word, unique identifier] | 26089 |
| preeclamp*.mp. [mp=title, abstract, original title, name of substance word, subject heading word, keyword heading word, protocol supplementary concept word, rare disease supplementary concept word, unique identifier] | 12721 |
| Hypertension, Pregnancy-Induced/ | 1711 |
| (gestational and hypertensi*).mp. [mp=title, abstract, original title, name of substance word, subject heading word, keyword heading word, protocol supplementary concept word, rare disease supplementary concept word, unique identifier] | 6056 |
| Postpartum Hemorrhage/ | 4842 |
| (postpartum and hemorrhag*).mp. [mp=title, abstract, original title, name of substance word, subject heading word, keyword heading word, protocol supplementary concept word, rare disease supplementary concept word, unique identifier] | 6438 |
| (postpartum and haemorrhag*).mp. [mp=title, abstract, original title, name of substance word, subject heading word, keyword heading word, protocol supplementary concept word, rare disease supplementary concept word, unique identifier] | 1573 |
| obstetric labor, premature/ or premature birth/ | 18496 |
| (preterm or pre-term).mp. [mp=title, abstract, original title, name of substance word, subject heading word, keyword heading word, protocol supplementary concept word, rare disease supplementary concept word, unique identifier] | 44753 |
| (birth or labor or labour or deliver*).mp. [mp=title, abstract, original title, name of substance word, subject heading word, keyword heading word, protocol supplementary concept word, rare disease supplementary concept word, unique identifier] | 730110 |
| 24 and 25 | 32432 |
| cesarean section/ or extraction, obstetrical/ or vacuum extraction, obstetrical/ or labor, induced/ | 43512 |
| (cesar* or caesar*).mp. [mp=title, abstract, original title, name of substance word, subject heading word, keyword heading word, protocol supplementary concept word, rare disease supplementary concept word, unique identifier] | 52780 |
| (induc* or instrument* or vacuum).mp. [mp=title, abstract, original title, name of substance word, subject heading word, keyword heading word, protocol supplementary concept word, rare disease supplementary concept word, unique identifier] | 2460462 |
| 25 and 29 | 95638 |
| exp Resuscitation/ | 74306 |
| exp thromboembolism/ or exp thrombosis/ | 142278 |
| Intensive Care Units/ | 36416 |
| Pregnancy/ | 702831 |
| 31 or 32 or 33 | 247846 |
| 34 and 35 | 8272 |
| (resusc* or thrombo* or intensive care or (high and depend*)).mp. [mp=title, abstract, original title, name of substance word, subject heading word, keyword heading word, protocol supplementary concept word, rare disease supplementary concept word, unique identifier] | 763789 |
| pregnan*.mp. [mp=title, abstract, original title, name of substance word, subject heading word, keyword heading word, protocol supplementary concept word, rare disease supplementary concept word, unique identifier] | 775673 |
| 37 and 38 | 30807 |
| failed instrumental delivery.mp. [mp=title, abstract, original title, name of substance word, subject heading word, keyword heading word, protocol supplementary concept word, rare disease supplementary concept word, unique identifier] | 17 |
| Episiotomy/ | 1709 |
| episiotomy.mp. | 2330 |
| anal sphincter injury.mp. | 162 |
| (third or fourth).mp. [mp=title, abstract, original title, name of substance word, subject heading word, keyword heading word, protocol supplementary concept word, rare disease supplementary concept word, unique identifier] | 379406 |
| degree tears.mp. | 120 |
| 44 and 45 | 103 |
| 12 or 13 or 14 or 15 or 16 or 17 or 18 or 19 or 20 or 21 or 22 or 23 or 26 or 27 or 28 or 30 or 36 or 39 or 40 or 41 or 42 or 43 or 46 | 236364 |
| exp Infant, Low Birth Weight/ | 26757 |
| low birth weight.mp. [mp=title, abstract, original title, name of substance word, subject heading word, keyword heading word, protocol supplementary concept word, rare disease supplementary concept word, unique identifier] | 30133 |
| small for gestational age.mp. [mp=title, abstract, original title, name of substance word, subject heading word, keyword heading word, protocol supplementary concept word, rare disease supplementary concept word, unique identifier] | 8085 |
| exp Birth Weight/ | 34313 |
| large for gestational age.mp. [mp=title, abstract, original title, name of substance word, subject heading word, keyword heading word, protocol supplementary concept word, rare disease supplementary concept word, unique identifier] | 1111 |
| Congenital Hyperinsulinism/ | 303 |
| ((hypoglycem* or hypoglycaem*) and neonat*).mp. [mp=title, abstract, original title, name of substance word, subject heading word, keyword heading word, protocol supplementary concept word, rare disease supplementary concept word, unique identifier] | 3013 |
| Fetal Death/ | 22997 |
| Respiratory Distress Syndrome, Newborn/ | 11479 |
| (fet* or foet* or neonat*).mp. [mp=title, abstract, original title, name of substance word, subject heading word, keyword heading word, protocol supplementary concept word, rare disease supplementary concept word, unique identifier] | 548601 |
| (respiratory distress or death or intensive care).mp. [mp=title, abstract, original title, name of substance word, subject heading word, keyword heading word, protocol supplementary concept word, rare disease supplementary concept word, unique identifier] | 660039 |
| 57 and 58 | 74044 |
| Intensive Care Units, Neonatal/ | 9922 |
| shoulder dystocia.mp. [mp=title, abstract, original title, name of substance word, subject heading word, keyword heading word, protocol supplementary concept word, rare disease supplementary concept word, unique identifier] | 932 |
| perinatal complication.mp. [mp=title, abstract, original title, name of substance word, subject heading word, keyword heading word, protocol supplementary concept word, rare disease supplementary concept word, unique identifier] | 46 |
| birth trauma.mp. [mp=title, abstract, original title, name of substance word, subject heading word, keyword heading word, protocol supplementary concept word, rare disease supplementary concept word, unique identifier] | 838 |
| umbilical cord ph.mp. [mp=title, abstract, original title, name of substance word, subject heading word, keyword heading word, protocol supplementary concept word, rare disease supplementary concept word, unique identifier] | 118 |
| neonatal adiposity.mp. | 38 |
| 48 or 49 or 50 or 51 or 52 or 53 or 54 or 55 or 56 or 59 or 60 or 61 or 62 or 63 or 64 or 65 | 134946 |
| 47 or 66 | 331748 |
| 11 and 67 | 6168 |
| limit 68 to (english language and female and humans and yr="1999 -Current") | 2726 |
