# Supplementary material for: Gestational weight gain across continents and ethnicity: systematic review and meta-analysis of maternal and infant outcomes in more than one million women
Source: BMC Med. 2018 Aug 31;16:153. doi: 10.1186/s12916-018-1128-1 (PMC6117916; doi:10.1186/s12916-018-1128-1)
Supplement: Supplementary file 2 — Additional methods. (DOCX 14 kb) [file 12916_2018_1128_MOESM2_ESM.docx]

**Additional file 2: Additional Methods**

Screening of search results

A trained clinician reviewer (RG) scanned the titles, abstract sections and keywords of every record retrieved by the search strategy in consultation with a highly experienced systematic reviewer (MM). Studies were selected and appraised by the reviewer in consultation with MM and clinical colleagues with evidence synthesis experience (HT) using study selection and appraisal criteria established *a priori*. Full articles were retrieved for all papers that met initial inclusion criteria, or if clarification was required beyond the abstract. In cases where selection was not clear, a second trained clinician reviewer appraised the paper (SA). Any discrepancies between the two appraisers was discussed with a third review author (MM).

Data extraction

Data were extracted from included studies using a specially developed data extraction form by two reviewers (RG, SA). Information collected included: type of study, study setting, study population, inclusion/exclusion criteria, outcomes measured and confounding factors. Missing data was obtained from the authors wherever possible. Any disagreement was resolved by discussion with an experienced biostatistician to reach a consensus (SR).

Data reanalysis request

Given the wide variation in classification of prepregnancy BMI categories and GWG categories, meaningful interpretation and meta-analysis was not possible. We therefore decided *a priori* to contact these authors to reanalyse and present data in a consistent, homogeneous format. For example, authors were requested to reclassify 1990 IOM GWG categories according to 2009 categories for data synthesis. If multiple weight gain groups within each BMI category were presented, authors were requested to reanalyse their data using 2009 categories.

Thirty one authors were contacted for data reanalysis and additional information, including the proportion of nulliparous women, proportion smoking in pregnancy, and mean maternal age for the meta-regression. This process involved email contact from the lead systematic review authors to the past senior study authors. Legal agreements for data sharing were prepared as well as authorship agreements where substantial reanalysis was required. Thirteen authors provided additional information and were included; eighteen did not provide this, of these three studies were still able to be included. Authors that were unable to reanalyse 1990 IOM data or correct multiple weight gain groups were excluded.
