# Supplementary material for: Gestational weight gain across continents and ethnicity: systematic review and meta-analysis of maternal and infant outcomes in more than one million women
Source: BMC Med. 2018 Aug 31;16:153. doi: 10.1186/s12916-018-1128-1 (PMC6117916; doi:10.1186/s12916-018-1128-1)
Supplement: Supplementary file 3 — Table S1. Descriptive characteristics of 23 included studies. (DOCX 27 kb) [file 12916_2018_1128_MOESM3_ESM.docx]

**Additional file 3: Table S1. Descriptive characteristics of 23 included studies**

| **Study, year,**  **country** | **Study period** | **Study design, sample size** | **Setting** | **Inclusion criteria** | **Exclusion criteria** | **Confounders in original analysis** | **Provided additional data or reanalysis** | **Data for meta-analysis:** |
| --- | --- | --- | --- | --- | --- | --- | --- | --- |
| Durst,  2016  US | 2000-2014 | Retrospective  5651 | University of Alabama, Birmingham | obese women; those delivering after 36 weeks gestation with documented weight in first trimester and within 10 days before delivery | NR | prior caesarean, age, race, parity, gestational age, payor status, tobacco use | no | Adjusted |
| Enomoto, 2016  Japan | 2013 | Retrospective  97157 | Japan Society of Obstetrics and Gynecology Registry system with 280 participating hospitals | singleton pregnancy, successful delivery occurring at gestational week 22 or later | women with hypertension of diabetes, history of cervical conization, who delivered a newborn with congenital anomalies, missing data | maternal age, height, parity and additional adjusting for clustering of deliveries by hospitals | no | Adjusted |
| Hung,  2016  Taiwan | 2009-2015 | Retrospective  10973 | Taipei Chang Gung Memorial Hospital | singleton pregnancy after 37 weeks gestation (cohort 2) | women with pregestational diabetes and hypertension, multiple gestations, fetal chromosomal or structural anomalies, fetal demise | maternal age, parity, prior fetal death, prior preterm birth, conception methods, genetic amniocentesis, smoking in pregnancy, group B strep colonization, fetal sex, epidural | no | Adjusted |
| Xiongᵉ, 2016  China | 2012-2013 | Prospective  57891 | Hospitals and community centres | singleton, live-born, term pregnancies | women with diabetes, hypertension, heart disease before or during pregnancy or those with missing height and weight data, women who delivered a stillborn infant or infant with birth defects | maternal age, education, parity, fetal sex, birth weight | no | Adjusted |

**Table 1. Descriptive characteristics of 23 included studies (continued)**

| **Study, year, country** | **Study period** | **Study design, sample size** | **Setting** | **Inclusion criteria** | **Exclusion criteria** | **Confounders in original analysis** | **Provided additional data or reanalysis** | **Data for meta-analysis:** |
| --- | --- | --- | --- | --- | --- | --- | --- | --- |
| Bogaerts, 2015  Belgium | 2009-2011 | Retrospective  18053 | Flemish study center for perinatal epidemiology | singleton, live births | GW loss >45 kg, GWG > 60kg, extreme prepregnancy weight/height and weight at delivery | parity, maternal age, gestational age | yes | crude |
| Shin,  2015  US | 2004-2011 | Retrospective 219868 | Pregnancy risk assessment monitoring system (PRAMS) | live births | missing prepregnancy BMI, GWG, preexisting DM and outcomes | maternal age, race, education, income, gestational age, WIC participation, smoking | yes | crude |
| Wenᵉ, 2015  China | 2009-2013 | Retrospective  13776 | Jishuitan Hospital | singleton, age 18-40, normal prepregnancy BMI, GWG ≤ 16kg, primipara | GWG > 16kg, any DM, HT, severe congenital anomalies, missing data on BMI, GWG, birth weight or pregnancy outcomes | income, maternal education, occupation, weight gain advice, residential area | no | adjusted |
| Yangᵉ, 2015  China | 2011-2013 | Prospective  85765 | Wuah Women and Children Health Care Center | singleton, live birth, gestational age ≥ 28 wk | NR | maternal age, maternal education, infant gender (provided crude and adjusted) | no | crude |
| Badon, 2014  US | 2000-2006 | Prospective  5297 | North American Field Centers, HAPO | pregnant women < 31 weeks gestation | age < 18, multiple pregnancy, previous diabetes, diabetes in pregnancy | gender, race, parity, study centre, maternal age, OGTT z score sum, alcohol use, smoking, family history of diabetes, hospitalisation pre delivery gestational age at last prenatal weight and OGTT mean arterial pressure at OGTT, maternal height | no | adjusted |
| Chihara, 2014  US | 2003-2005 | Retrospective  19130 | Hawaii's special supplemental program for women, infants and children (WIC) | NR | no prenatal record, gestational age <20 or >44 wk, multiple births, missing GWG, birthweight | maternal age, education, race/ethnicity, marital status, smoking status, parity | no | adjusted |

**Table 1. Descriptive characteristics of 23 included studies (continued)**

| **Study, year, country** | **Study period** | **Study design, sample size** | **Setting** | **Inclusion criteria** | **Exclusion criteria** | **Confounders in original analysis** | **Provided additional data or reanalysis** | **Data for meta-analysis:** |
| --- | --- | --- | --- | --- | --- | --- | --- | --- |
| Haugenᶜ, 2014  Norway | 1999-2008 | Prospective  56082 | Norwegian Mother and Child cohort study | prepregnancy weight and height, weight at delivery and 6 months post partum | gestation < 37 or > 42 wk, GWG < -30kg or > 50kg, age < 18 years, women with 2nd or 3rd participation in study | maternal age, maternal height, maternal education, gestational length, smoking, diabetes | yes | Crude |
| Leeᵈ, 2014  Korea | 2010-2012 | Retrospective  16297 | Single medical centre | singleton, live births | pre-existing medical conditions (diabetes and HT) | maternal age, parity | yes | crude |
| Swank, 2014  US | 2007 | Retrospective  1034 | Californian birth certificate data | singleton, live birth, gestation 24-42 (+6) wk | unknown prepregnancy BMI | maternal age, parity, race, hypertension, pregestational diabetes | yes | crude |
| Black, 2013  US | 2005-2010 | Retrospective  9835 | Kaiser Permanente Southern California | singleton, live birth gestation ≥ 20 wk | those requiring treatment for GDM | maternal age, race/ethnicity, parity, infant sex, presence of PE/E | yes | crude |
| Kominiarekᵇ, 2013  US | 2002- 2008 | Retrospective  21020 | 12 institutions (19 hospitals) | BMI ≥ 30 kg/m², singleton, live birth, ≥ 37 wk, known GWG | weight loss > 20kg, weight gain > 50kg | age, race/ethnicity, marital status, insurance, parity, smoking, gestational age | yes | adjusted |
| Liᵅ, 2013  China | 2009-2011 | Retrospective  33973 | Tianjin Women and Children's Health Center | mother-child pairs with information and clinical measurements | multiple births, stillbirths, multiparous women, missing variables | maternal age, maternal height, maternal education, smoking, family income, maternal occupation,  gestational age | yes | crude |
| Di Benedetto, 2012  Italy | 2004-2009 | Retrospective  2225 | University Hospital | Caucasian women, had glucose challenge test | gestation < 37 weeks, twin pregnancy, glucose intolerance in pregnacy, missing delivery information | gestational age at delivery, glycaemia | yes | crude |
| Moore Simas, 2012  US | 2006-2010 | Retrospective  11203 | University Hospital | singleton, live birth | congenital anomaly, missing prepregnancy weight, height, GWG, unknown neonate gender or weight, gestation < 22 wk or > 44 wk | both crude and adjusted. marital status, race, parity, smoking, diabetes, hypertension | yes | crude |

**Table 1. Descriptive characteristics of 23 included studies (continued)**

| **Study, year, country** | **Study period** | **Study design, sample size** | **Setting** | **Inclusion criteria** | **Exclusion criteria** | **Confounders in original analysis** | **Provided additional data or reanalysis** | **Data for meta-analysis:** |
| --- | --- | --- | --- | --- | --- | --- | --- | --- |
| Blomberg, 2011  Sweden | 1993-2008 | Retrospective  46595 | Swedish Medical birth registry | BMI ≥ 30 kg/m², singleton, live birth, ≥ 37 wk | extreme GWG or GW loss | maternal age, parity, smoking | no | adjusted |
| J Parkᵈ 2011  Korea | 2005-2007 | Retrospective  2311 | University Hospital | live births, gestation 28-42 weeks | missing prepregnancy BMI, hypertension, diabetes, twin pregnancy, congenital anomaly, previous caesarean | both crude and adjusted. BMI, smoking, parity, education, husbnad's education, gestational age, gestational diabetes | yes | crude |
| S Park, 2011  US | 2004-2007 | Retrospective  560672 | Florida birth certificate data | singleton, live birth, gestational 37-41 wk, age 18-40 years | chronic diabetes, chronic hypertension, missing information for BMI, GWG, LGA or SGA status | maternal age, parity, gestational age, education, smoking, WIC program participation, total number of prenatal visits, infant sex, infant birth year | yes | crude |
| Vesco, 2011  US | 2000-2005 | Retrospective  2080 | Kaiser Permanente group practice | prepregnancy weight, delivery weight, height | diabetes (gestational and pregestational), hypertension | age, BMI, gestation, race, parity smoking, Medicaid (provided crude and adjusted) | no | crude |
| Rode, 2007  Denmark | 1996-1998 | Prospective  2248 | University Hospital | age > 18 years, Danish speaking, no alcohol or drug abuse, completed both questionnaires | multiple gestations, gestational age < 37 weeks, missing infant birth weight | smoking status | yes | crude |

| **Key** |  |  |  |  |  |
| --- | --- | --- | --- | --- | --- |
| a | data according to both Chinese and WHO BMI categories (Chinese reported here) | | | | |
| b | sample size changed when provided additional data, OR not recalculated | | | | |
| c | sample size changed when provided additional data | | | |  |
| d | data according to both Korean and WHO BMI categories (Korean reported here) | | | |  |
| e | data according to Chinese BMI categories | | |  |  |
| NR | not reported | |  |  |  |
