# Supplementary material for: Gestational weight gain across continents and ethnicity: systematic review and meta-analysis of maternal and infant outcomes in more than one million women
Source: BMC Med. 2018 Aug 31;16:153. doi: 10.1186/s12916-018-1128-1 (PMC6117916; doi:10.1186/s12916-018-1128-1)
Supplement: Supplementary file 4 — Figure S1. Summary of pooled OR for the association between gestational weight gain below and above guidelines for adverse outcomes. (DOCX 220 kb) [file 12916_2018_1128_MOESM4_ESM.docx]

**Additional file 4: Summary of pooled OR for the association between gestational weight gain below and above guidelines for adverse outcomes**

Figure 1a. Small for gestational age (SGA): GWG below guidelines

Decreased odds of outcome Increased odds of outcome

Reference group = women with recommended weight gain in each BMI group

Figure 1b. Small for gestational age (SGA): GWG above guidelines

Decreased odds of outcome Increased odds of outcome

Reference group = women with recommended weight gain in each BMI group

Figure 1c. Preterm birth: GWG below guidelines

Decreased odds of outcome Increased odds of outcome

Reference group = women with recommended weight gain in each BMI group

Figure 1d. Preterm birth: GWG above guidelines

Decreased odds of outcome Increased odds of outcome

Reference group = women with recommended weight gain in each BMI group

Figure 1e. Large for gestational age (LGA): GWG below guidelines

Decreased odds of outcome Increased odds of outcome

Reference group = women with recommended weight gain in each BMI group

Figure 1f. Large for gestational age (LGA): GWG above guidelines

Decreased odds of outcome Increased odds of outcome

Reference group = women with recommended weight gain in each BMI group

Figure 1g. Macrosomia: GWG below guidelines

Decreased odds of outcome Increased odds of outcome

Reference group = women with recommended weight gain in each BMI group

Figure 1h. Macrosomia: GWG above guidelines

Decreased odds of outcome Increased odds of outcome

Reference group = women with recommended weight gain in each BMI group

Figure 1i. Caesarean delivery: GWG below guidelines

Decreased odds of outcome Increased odds of outcome

Reference group = women with recommended weight gain in each BMI group

Figure 1j. Caesarean delivery: GWG above guidelines

Decreased odds of outcome Increased odds of outcome

Reference group = women with recommended weight gain in each BMI group

Decreased odds of outcome Increased odds of outcome

Reference group = women with recommended weight gain in each BMI group
