# Supplementary material for: Gestational weight gain across continents and ethnicity: systematic review and meta-analysis of maternal and infant outcomes in more than one million women
Source: BMC Med. 2018 Aug 31;16:153. doi: 10.1186/s12916-018-1128-1 (PMC6117916; doi:10.1186/s12916-018-1128-1)
Supplement: Supplementary file 5 — Table S2. Body mass index at onset of pregnancy for Asian studies. (DOCX 17 kb) [file 12916_2018_1128_MOESM5_ESM.docx]

**Additional file 5: Table S2.** Body mass index at onset of pregnancy for Asian studies

Body mass index at onset of pregnancy, n (%)

| Including all studies | | | | | |
| --- | --- | --- | --- | --- | --- |
| Country | Underweight | Normal weight | Overweight | Obese | Total |
| China | 28330 (15) | 145721 (76) | 14905 (8) | 2449 (1) | 191405 |
| Korea | 3040 (16) | 11979 (64) | 1937 (10) | 1652 (9) | 18608 |
| Japan | 17724 (18) | 69126 (71) | 7502 (8) | 2805 (3) | 97157 |
| Taiwan | 1556 (14) | 8247 (75) | 961 (9) | 209 (2) | 10973 |
| Excluding studies that selected for normal weight only ([16](#_ENREF_16)) (study from China) | | | | | |
| China | 28330 (16) | 131945 (74) | 14905 (8) | 2449 (1) | 177629 |
| Korea | 3040 (16) | 11979 (64) | 1937 (10) | 1652 (9) | 18608 |
| Japan | 17724 (18) | 69126 (71) | 7502 (8) | 2805 (3) | 97157 |
| Taiwan | 1556 (14) | 8247 (75) | 961 (9) | 209 (2) | 10973 |
