# Supplementary material for: Gestational weight gain across continents and ethnicity: systematic review and meta-analysis of maternal and infant outcomes in more than one million women
Source: BMC Med. 2018 Aug 31;16:153. doi: 10.1186/s12916-018-1128-1 (PMC6117916; doi:10.1186/s12916-018-1128-1)
Supplement: Supplementary file 6 — Table S3. Gestational weight gain during pregnancy for Asian studies. (DOCX 14 kb) [file 12916_2018_1128_MOESM6_ESM.docx]

**Additional file 6: Table S3.** Gestational weight gain during pregnancy for Asian studies

Gestational weight gain during pregnancy, n (%)

| Including all studies | | | | |
| --- | --- | --- | --- | --- |
| Region | Below guidelines | Within guidelines | Above guidelines | Total |
| China | 28748 (15) | 61668 (32) | 100989 (53) | 191405 |
| Korea | 4983 (27) | 7989 (43) | 5636 (30) | 18608 |
| Japan | 62005 (64) | 28281 (29) | 6871 (7) | 97157 |
| Taiwan | 3156 (29) | 4948 (45) | 2869 (26) | 10973 |
