# Supplementary material for: Gestational weight gain across continents and ethnicity: systematic review and meta-analysis of maternal and infant outcomes in more than one million women
Source: BMC Med. 2018 Aug 31;16:153. doi: 10.1186/s12916-018-1128-1 (PMC6117916; doi:10.1186/s12916-018-1128-1)
Supplement: Supplementary file 7 — Figure S2. Asian subgroup analysis: studies using local BMI categories (China, Korea) vs WHO BMI categories (Japan, Taiwan). Summary of pooled OR for the association between gestational weight gain below and above guidelines for adverse outcomes. (DOCX 106 kb) [file 12916_2018_1128_MOESM7_ESM.docx]

**Additional file 7: Asian subgroup analysis:** studies using local BMI categories (China, Korea) vs WHO BMI categories (Japan, Taiwan): summary of pooled OR for the association between gestational weight gain below and above guidelines for adverse outcomes

Figure 2a. Small for gestational age (SGA): GWG below guidelines

Figure 2b. Small for gestational age (SGA): GWG above guidelines

Decreased odds of outcome Increased odds of outcome

Reference group = women with recommended weight gain in each BMI group

Figure 2c. Large for gestational age (LGA): GWG below guidelines

Decreased odds of outcome Increased odds of outcome

Reference group = women with recommended weight gain in each BMI group

Figure 2d. Large for gestational age (LGA): GWG above guidelines

Decreased odds of outcome Increased odds of outcome

Reference group = women with recommended weight gain in each BMI group

Figure 2e. Macrosomia: GWG below guidelines

Decreased odds of outcome Increased odds of outcome

Reference group = women with recommended weight gain in each BMI group

Figure 2f. Macrosomia: GWG above guidelines

Decreased odds of outcome Increased odds of outcome

Reference group = women with recommended weight gain in each BMI group

Decreased odds of outcome Increased odds of outcome

Reference group = women with recommended weight gain in each BMI group

Figure 2g. Caesarean section: GWG below guidelines

Decreased odds of outcome Increased odds of outcome

Reference group = women with recommended weight gain in each BMI group

Figure 2h. Caesarean section: GWG above guidelines

Decreased odds of outcome Increased odds of outcome

Reference group = women with recommended weight gain in each BMI group
