# Supplementary material for: Gestational weight gain across continents and ethnicity: systematic review and meta-analysis of maternal and infant outcomes in more than one million women
Source: BMC Med. 2018 Aug 31;16:153. doi: 10.1186/s12916-018-1128-1 (PMC6117916; doi:10.1186/s12916-018-1128-1)
Supplement: Supplementary file 8 — Table S4. Meta-regression. (DOCX 21 kb) [file 12916_2018_1128_MOESM8_ESM.docx]

**Additional file 8: Table S4. Metaregression***

Table 4a. Small for gestational age (SGA): GWG below guidelines for US/Europe

| Variable  (log OR) | Coefficient | p-value | Lower CI | Upper CI | I² (%) | p-value |
| --- | --- | --- | --- | --- | --- | --- |
| Smoking (yes) | -0.0135231 | 0.056 | -0.0274272 | 0.000381 | 0.10 | 0.0000 |
| Mean maternal age (years) | -0.0781185 | 0.000 | -0.1061379 | -0.050099 |  |  |
| Nulliparous (yes) | 0.0196059 | 0.000 | 0.0151648 | 0.0240469 |  |  |

Table 4b. Small for gestational age (SGA): GWG below guidelines for Asia

| Variable  (log OR) | Coefficient | p-value | Lower CI | Upper CI | I² (%) | p-value |
| --- | --- | --- | --- | --- | --- | --- |
| Smoking (yes) | 0.1964278 | 0.369 | -0.342288 | 0.7351436 | 36.44 | 0.5132 |
| Mean maternal age (years) | -0.1933659 | 0.604 | -1.148952 | 0.7622198 |  |  |
| Nulliparous (yes) | 0.0111027 | 0.794 | -0.0995421 | 0.1217475 |  |  |

Table 4c. Small for gestational age (SGA): GWG above guidelines for US/Europe

| Variable  (log OR) | Coefficient | p-value | Lower CI | Upper CI | I² (%) | p-value |
| --- | --- | --- | --- | --- | --- | --- |
| Smoking (yes) | -0.0104975 | 0.414 | -0.0375238 | 0.0165287 | 62.11 | 0.3786 |
| Mean maternal age (years) | -0.0052822 | 0.850 | -0.0647809 | 0.0542165 |  |  |
| Nulliparous (yes) | -0.0064535 | 0.203 | -0.0168867 | 0.0039797 |  |  |

Table 4d. Large for gestational age (LGA): GWG below guidelines for US/Europe

| Variable  (log OR) | Coefficient | p-value | Lower CI | Upper CI | I² (%) | p-value |
| --- | --- | --- | --- | --- | --- | --- |
| Smoking (yes) | -0.0197174 | 0.192 | -0.0509173 | 0.0114826 | 0.00 | 0.0015 |
| Mean maternal age (years) | 0.0738492 | 0.021 | 0.0137145 | 0.1339839 |  |  |
| Nulliparous (yes) | -0.0202366 | 0.000 | -0.0289771 | -0.0114961 |  |  |

Table 4e. Large for gestational age (LGA): GWG below guidelines for Asia

| Variable  (log OR) | Coefficient | p-value | Lower CI | Upper CI | I² (%) | p-value |
| --- | --- | --- | --- | --- | --- | --- |
| Smoking (yes) | -0.0487653 | 0.814 | -0.5884467 | 0.4909161 | 0.0 | 0.6590 |
| Mean maternal age (years) | 0.1316793 | 0.712 | -0.7915934 | 1.054952 |  |  |
| Nulliparous (yes) | 0.0074661 | 0.853 | -0.0974589 | 0.1123911 |  |  |

Table 4f. Large for gestational age (LGA): GWG above guidelines for US/Europe

| Variable  (log OR) | Coefficient | p-value | Lower CI | Upper CI | I² (%) | p-value |
| --- | --- | --- | --- | --- | --- | --- |
| Smoking (yes) | -0.0169545 | 0.244 | -0.0471117 | 0.0132027 | 68.31 | 0.0890 |
| Mean maternal age (years) | -0.0543429 | 0.084 | -0.1170754 | 0.0083897 |  |  |
| Nulliparous (yes) | 0.0134203 | 0.025 | 0.0020001 | 0.0248405 |  |  |

Table 4g. Large for gestational age (LGA): GWG above guidelines for Asia

| Variable  (log OR) | Coefficient | p-value | Lower CI | Upper CI | I² (%) | p-value |
| --- | --- | --- | --- | --- | --- | --- |
| Smoking (yes) | 0.0063775 | 0.839 | -0.0704408 | 0.0831958 | 28.81 | 0.2477 |
| Mean maternal age (years) | -0.373568 | 0.134 | -0.9114196 | 0.1642835 |  |  |
| Nulliparous (yes) | -0.0246403 | 0.215 | -0.0692884 | 0.0200078 |  |  |

Table 4h. Macrosomia: GWG below guidelines for Asia

| Variable  (log OR) | Coefficient | p-value | Lower CI | Upper CI | I² (%) | p-value |
| --- | --- | --- | --- | --- | --- | --- |
| Smoking (yes) | -0.0100196 | 0.985 | -2.107265 | 2.087226 | 0.00 | 0.9691 |
| Mean maternal age (years) | 0.096737 | 0.810 | -1.427319 | 1.620793 |  |  |
| Nulliparous (yes) | 0.0087369 | 0.932 | -0.3801903 | 0.3976642 |  |  |

Table 4i. Macrosomia: GWG above guidelines for US/Europe

| Variable  (log OR) | Coefficient | p-value | Lower CI | Upper CI | I² | p-value |
| --- | --- | --- | --- | --- | --- | --- |
| Smoking (yes) | -0.0139481 | 0.287 | -0.0414042 | 0.013508 | 56.87 | 0.4430 |
| Mean maternal age (years) | -0.0744533 | 0.363 | -0.2471216 | 0.0982151 |  |  |
| Nulliparous (yes) | -0.0089618 | 0.529 | -0.039307 | 0.0213833 |  |  |

Table 4j. Macrosomia: GWG above guidelines for Asia

| Variable  (log OR) | Coefficient | p-value | Lower CI | Upper CI | I² (%) | p-value |
| --- | --- | --- | --- | --- | --- | --- |
| Smoking (yes) | 0.1268428 | 0.615 | -0.5955475 | 0.8492331 | 47.34 | 0.7675 |
| Mean maternal age (years) | -0.2914988 | 0.390 | -1.217734 | 0.6347363 |  |  |
| Nulliparous (yes) | 0.0003095 | 0.994 | -0.1157701 | 0.1163891 |  |  |

Table 4k. Caesarean section: GWG below guidelines for Asia

| Variable  (log OR) | Coefficient | p-value | Lower CI | Upper CI | I² | p-value |
| --- | --- | --- | --- | --- | --- | --- |
| Smoking (yes) | 0.1242494 | 0.461 | -0.2990494 | 0.5475481 | 49.89 | 0.6020 |
| Mean maternal age (years) | -0.3204323 | 0.301 | -1.069926 | 0.4290615 |  |  |
| Nulliparous (yes) | -0.0060738 | 0.853 | -0.0912339 | 0.0790863 |  |  |

Table 4l. Caesarean section: GWG over guidelines for Asia

| Variable  (log OR) | Coefficient | p-value | Lower CI | Upper CI | I² | p-value |
| --- | --- | --- | --- | --- | --- | --- |
| Smoking (yes) | -0.1295935 | 0.288 | -0.4235213 | 0.1643342 | 25.66 | 0.4990 |
| Mean maternal age (years) | 0.120274 | 0.328 | -0.4205241 | 0.179976 |  |  |
| Nulliparous (yes) | -0.030367 | 0.186 | -0.0832446 | 0.0225107 |  |  |

*REML estimate of between-study variance % residual variation due to heterogeneity. Proportion of between-study variance is explained using the Joint test for all covariates with Knapp-Hartung modification
