# Supplementary material for: Gestational weight gain across continents and ethnicity: systematic review and meta-analysis of maternal and infant outcomes in more than one million women
Source: BMC Med. 2018 Aug 31;16:153. doi: 10.1186/s12916-018-1128-1 (PMC6117916; doi:10.1186/s12916-018-1128-1)
Supplement: Supplementary file 9 — Figure S3. Publication bias. (DOCX 53 kb) [file 12916_2018_1128_MOESM9_ESM.docx]

**Additional file 9: Figure S3. Publication bias**

3a. SGA: GWG below guidelines for US/Europe

3b. SGA: GWG over guidelines for US/Europe

3c. LGA: GWG under guidelines for US/Europe

3d. LGA: GWG under guidelines for Asia

3e. LGA: GWG over guidelines for US/Europe

3f. LGA: GWG over guidelines for Asia

3g. Caesarean section: GWG under guidelines for Asia

3h. Caesarean section: GWG over guidelines for Asia

3i. Macrosomia: GWG below guidelines for US/Europe

3j. Macrosomia: GWG below guidelines for Asia

3k. Macrosomia: GWG above guidelines for US/Europe

3l. Macrosomia: GWG above guidelines for Asia
