# Supplementary material for: Gestational weight gain across continents and ethnicity: systematic review and meta-analysis of maternal and infant outcomes in more than one million women
Source: BMC Med. 2018 Aug 31;16:153. doi: 10.1186/s12916-018-1128-1 (PMC6117916; doi:10.1186/s12916-018-1128-1)
Supplement: Supplementary file 10 — Table S5. Summary of risk of bias assessment. (DOCX 20 kb) [file 12916_2018_1128_MOESM10_ESM.docx]

**Additional file 10: Table S5. Summary of risk of bias assessment**

| **Study, year** | **Selection bias** Exposed cohort representative | **Detection bias** | | **Reporting bias** Free of selective outcome reporting | **Assessment of confounding in original analysis** | **Conflict of interest** | **Overall risk of bias** |
| --- | --- | --- | --- | --- | --- | --- | --- |
|  |  | Adequate exposure measures | Adequate outcome measures |  |  |  |  |
| Durst, 2016 | yes | yes | yes | yes | yes | no | low |
| Enomoto, 2016 | yes | NR | yes | yes | yes | no | low |
| Hung, 2016 | yes | yes | yes | yes | yes | no | low |
| Xiong, 2016 | yes | yes | yes | yes | yes | no | low |
| Bogaerts, 2015 | yes | yes | yes | yes | yes | no | low |
| Shin, 2015 | yes | yes | no (self reported) | yes | partially (did not adjust for parity) | no | moderate |
| Wen, 2015 | NR | yes | NR | partial (not all outcomes reported) | partially (did not adjust for required number of confounders) | no | moderate |
| Yang, 2015 | yes | yes | yes | yes | yes | no | low |
| Badon, 2014 | yes | yes | yes | yes | yes | no | low |
| Chihara, 2014 | yes | partial (self reported final weight) | no (self reported) | yes | yes | NR | moderate |
| Haugen, 2014 | yes | partial (self reported final weight) | yes | yes | yes | no | low |
| Lee, 2014 | NR | yes | yes | yes | yes | no | low |
| Swank, 2014 | yes | yes | yes | yes | yes | no | low |
| Black, 2013 | yes | yes | yes | yes | yes | no | low |
| Kominiarek, 2013 | yes | yes | yes | yes | yes | no | low |
| Li, 2013 | yes | yes | yes | yes | yes | no | low |
| Di Benedetto, 2012 | yes | yes | yes | yes | partially (did not adjust for parity) | no | low |
| Moore Simas, 2012 | yes | partial (some self reported final weight) | yes | yes | yes | no | low |

**Table S5. Summary of risk of bias assessment (continued)**

| **Study, year** | **Selection bias** Exposed cohort representative | **Detection bias** | | **Reporting bias** Free of selective outcome reporting | **Assessment of confounding in original analysis** | **Conflict of interest** | **Overall risk of bias** |
| --- | --- | --- | --- | --- | --- | --- | --- |
|  |  | Adequate exposure measures | Adequate outcome measures |  |  |  |  |
| Blomberg, 2011 | yes | yes | yes | yes | yes | no | low |
| J Park, 2011 | yes | yes | yes | partial (not all outcomes reported) | yes | NR | low |
| S Park, 2011 | partial | NR | yes | yes | yes | NR | low |
| Vesco, 2011 | yes | yes | yes | yes | yes | no | low |
| Rode, 2007 | NR | partial (self reported final weight) | yes | yes | partially (did not adjust for parity) | NR | low |

NR = not reported
